# Single-cell SNP analyses and interpretations based on RNA-Seq data for colon cancer research

**DOI:** 10.1038/srep34420

**Published:** 2016-09-28

**Authors:** Jiahuan Chen, Qian Zhou, Yangfan Wang, Kang Ning

**Affiliations:** 1 Key Laboratory of Molecular Biophysics of the Ministry of Education, Hubei Key Laboratory of Bioinformatics and Molecular-imaging, Department of Bioinformatics and Systems Biology, College of Life Science and Technology, Huazhong University of Science and Technology, Wuhan, Hubei, 430074, China; 2 Bioinformatics Group of the Single Cell Center, Shandong Key Laboratory of Energy Genetics and CAS Key Laboratory of Biofuels, Qingdao Institute of Bioenergy and Bioprocess Technology, Chinese Academy of Sciences, Qingdao, Shandong, 266101, China; 3 College of Marine Life, Ocean University of China, Qingdao, Shandong, 266000, China; 4 Shanghai Center for Systems Biomedicine, Shanghai Jiao Tong University, Shanghai, 200240, China

## Abstract

Single-cell sequencing is useful for illustrating the cellular heterogeneities inherent in many intricate biological systems, particularly in human cancer. However, owing to the difficulties in acquiring, amplifying and analyzing single-cell genetic material, obstacles remain for single-cell diversity assessments such as single nucleotide polymorphism (SNP) analyses, rendering biological interpretations of single-cell omics data elusive. We used RNA-Seq data from single-cell and bulk colon cancer samples to analyze the SNP profiles for both structural and functional comparisons. Colon cancer-related pathways with single-cell level SNP enrichment, including the TGF-β and p53 signaling pathways, were also investigated based on both their SNP enrichment patterns and gene expression. We also detected a certain number of fusion transcripts, which may promote tumorigenesis, at the single-cell level. Based on these results, single-cell analyses not only recapitulated the SNP analysis results from the bulk samples but also detected cell-to-cell and cell-to-bulk variations, thereby aiding in early diagnosis and in identifying the precise mechanisms underlying cancers at the single-cell level.

Colon cancer, one of the leading causes of cancer-related deaths worldwide^1^, has been widely studied^2^. Recent studies have already revealed important mechanisms underlying colon cancer pathogenesis^2^. A few oncogenes, some tumor-suppressor genes and a large number of related genes are mutated in a substantial fraction of colon cancer cases. The acquisition of multiple tumor-associated mutations in these genes initiates or drives the development of colon cancer^3^. Additionally, associated aberrant DNA methylation and chromosomal instability also dysregulate conserved signaling networks and disturb the regulation of cellular metabolism, proliferation, differentiation, and survival^3^. However, much work remains to be conducted to better identify and understand the genetic changes in colon cancer development, which is essential for the development of appropriate therapeutic strategies.

In recent years, with the advancement of next generation sequencing (NGS) technologies, great progress has been made in cancer genetic researches. These technologies provide us with not only immense quantities of data but also more detailed and accurate genetic information. However, the identification of genetic heterogeneity at the single-cell level, which is essential for reconstructing the evolutionary history of tumors and for revealing the mechanism of tumor occurrence and metastasis at the single-cell level^4^, may be averaged out in bulk sequencing^5^. This is the case even though the levels of some specific transcripts can vary by as much as 1000-fold^2^ between presumably equivalent cells, as measured by Fluorescence *In Situ* Hybridization (FISH). Moreover, rare mutations, which differ from the common mutations that are likely detected in most bulk samples, can be detected only in some single-cell samples. Therefore, the demand is growing for single-cell genetic profiling to accelerate the development of single-cell technologies. Single-cell sequencing is increasingly becoming the focus in many fields because of its ability to provide accurate measurements with a moderate number of sequencing reads and to recapitulate bulk complexity with a relatively large number of single cells, as well as its superiority in detecting single-cell heterogeneity^6^.

Although single-cell sequencing technology has continuously advanced cancer research, this novel technology still faces several obstacles and has much room for improvement^7^. In single-cell DNA sequencing, whole genome amplification (WGA) of such a small amount of DNA in an individual cell remains difficult owing to unregulated artificial errors, an inconsistent amplification ratio and lower coverage. These issues have led to SNP dropouts and false positives in recent studies^8^,^9^. The recently developed multiple annealing and looping-based amplification cycles (MALBAC) method^10^ has largely improved the uniformity across the genome^11^. In single-cell RNA sequencing, additional problems like full-length cDNA generation and low abundance transcript detection have hindered accurate analyses with higher resolution^11^,^12^,^13^. Additionally, the bioinformatic tools and algorithms designed for bulk samples have not been extensively evaluated in single-cell samples. Moreover, many of these tools do not account for the intrinsic problems originated from current single-cell amplification. Owing to these technical and analytical difficulties, only a few systematically generated single-cell genomic or transcriptomic data are available for routine omics interpretations. Therefore, this technology still faces difficulties in the systematic assessment of cell-level diversities and thus renders inaccurate interpretations from single-cell omics data.

In this study, we have collected RNA-Seq data sets from 96 single cells, 4 bulk samples of HCT116 cancer cells (samples were prepared as previously described^14^), and 1 bulk normal sigmoid colon sample. First, we used the single-cell RNA-Seq data to call SNPs using three SNP callers, analyzed the evolutional stress on Gene Ontology (GO) Slim terms, and compared the profiles of SNPs, which were enriched on chr11 and chr17, among the 83 selected single-cells. Second, by implementing GO analysis, SNP enrichments were shown in several GO Slim terms such as signal.transduction, while obvious cell heterogeneities were also observed. Third, we selected 175 cancer-related genes curated from previous studies and we detected that the SNPs were enriched in some of these genes in cancer-related pathways, even though not all of them were consistently identified. In colon cancer-related pathways such as the TGF-β and p53 signaling pathways, we found a list of mutated genes, some of which showed SNP enrichments. We speculated that these cancer-related genes and pathways might play key roles in the occurrence and metastasis of colon cancer. Finally, to examine the differences in the identified SNPs based on single-cell and bulk samples, we performed SNP analyses on RNA-Seq data using bulk cancer and normal samples. By comparing these results at the single-cell and bulk levels, it was clear that single-cell analyses were not only capable of recapitulating the bulk analyses results such as SNP profiles, cancer-related genes and pathways, but also specialized in detecting some variances and genetic features such as single-cell specific variations in *BMP7, CYCS and* some 14-3-3 protein-encoding genes in the subpopulations of single cells. Additionally, we searched for fusion genes in the single-cell sets and found specific transcripts that might have potential to accelerate tumor progression. Together, these comparisons revealed the globally consistent but locally different cell-to-cell and cell-to-bulk SNP variations.

## Results and Discussions

### General SNP calling results using different SNP callers

All of the 96 single cells have their transcripts sequenced on a HiSeq lane, with each single-cell obtained around 2 million raw reads on average, and the SNP calling analyses were performed based on these reads.

We obtained three sets of SNP calling results generated by The Genome Analysis Toolkit (GATK)^15^, SAMTools^16^ and Genotype Model Selection (GeMS)^17^, respectively. We implemented strict criteria to filter potential false positive SNPs (please refer to the Materials and Methods for details). On average, the GATK obtained 823.34 SNPs, SAMTools obtained 238.13 SNPs, and GeMS obtained 236.06 SNPs for each cell. While SNPs from GATK set targeted 761 genes, those detected by SAMTools and GeMS only targeted less than 60 genes, most of which were the same. Here we have selected the GATK (as the default method) and GeMS (as a Supplementary method) SNP calling results for follow-up analyses.

When associating SNP-Freq_g_, (defined by Formula (3) in the Materials and Methods) with coefficient of variation (CV) of SNP-Freq_g_, a strong negative correlation was presented (Supplementary Fig. 1). Similar result was observed when we tried to probe the association between gene expression and the corresponding CV (Supplementary Fig. 2). Across the dynamic range of expression or SNP-Freq_g_ of mutated transcripts, smaller values seemed to have larger variations, partially due to the technical noise. We also analyzed possible correlations of CV between expression and SNP-Freq_g_, and the result suggsted that there was no linear correlation between them (Supplementary Fig. 3). We also search for possible correlations between Fano factor and SNP-Freq_g_ and its correlations with expression. Here, we didn’t observe even a moderate statistical correlation between Fano and SNP-Freq_g_ (Supplementary Fig. 4), whereas a positive correlation was observed between Fano and expression (Supplementary Fig. 5).

Furthermore, evolutionary stress was observed under the assumption that each single-cell had undergone largely independent evolution (Supplementary Fig. 6, please refer to the Materials and Methods for details). Positive selections^18^ occurred in the GO Slim terms like homeostatic.process and signal.transduction. These positive selections of potential functions might help us to classify the process of tumor progression in a branching rather than a linear manner^18^. For these GO Slim terms with high Ka/Ks values (>1, positive selections), we considered the colon cancer cell line might go through a secondary tumor growth, and we speculated that the mutation frequencies might change dramatically during the culturing process. Additionally, we suggested that a stabilizing selection may account for the enrichment of GO Slim terms with negative selections such as response.to.stress. For instance, the culture environment likely forces the cell line to eliminate cells lacking certain mutations, allowing the population to outcompete and outgrow the non-mutated cells^18^. We also observed a negative correlation (*p* < 2.2e-16) between the Ka/Ks values of orthologous genes and their expressions (Supplementary Fig. 7). We hypothesized that larger Ka/Ks values for orthologs could reduce their expressions, as they might acquire mutations more frequently^19^.

### Comparison of SNP profiles and genomic structures of the selected single cells

**Figure 1:**
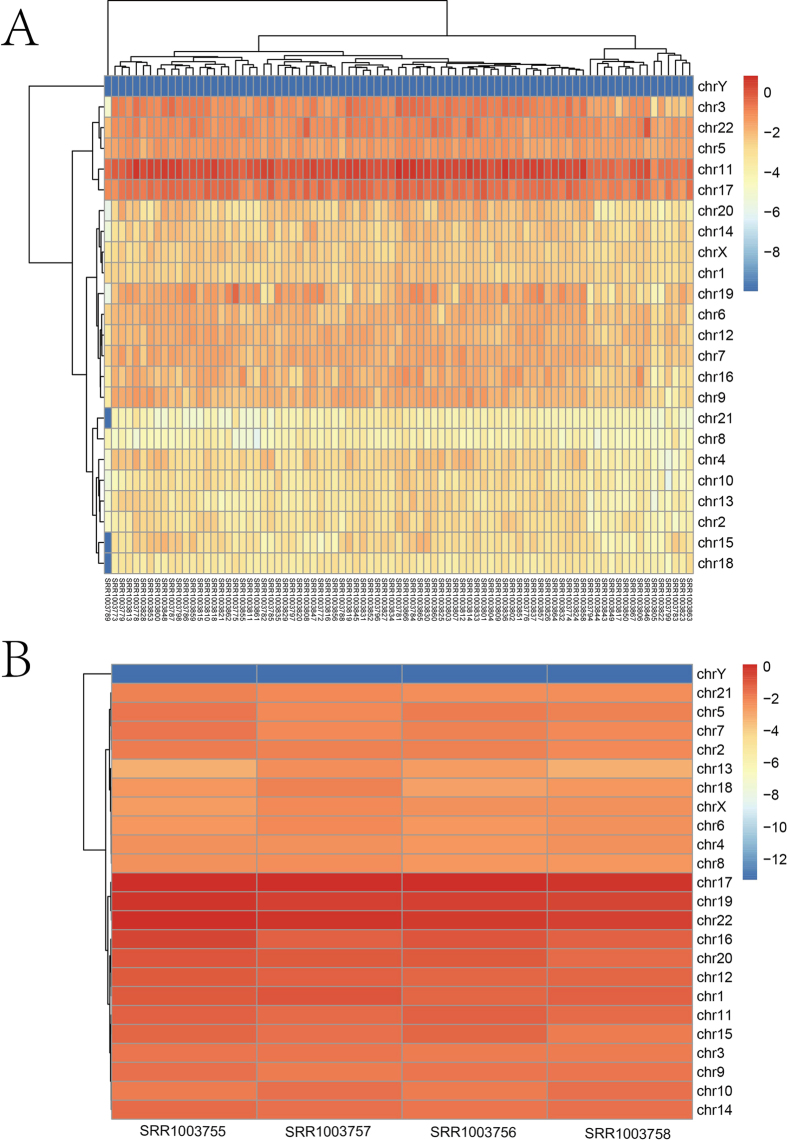
Heatmap of SNP enrichment on human chromosomes based on SNP calling results by the GATK. (**A**) Heatmap of the SNP-Freq_c_ (definition in Materials and Methods) on each chromosome for the 83 single-cell samples obtained by the GATK. (**B**) Heatmap of the SNP-Freq_c_ on each chromosome for the bulk (both cancer and normal) samples obtained by the GATK. The SNP enrichment on chromosomes for each single-cell and bulk (both cancer and normal) sample was shown in the heatmaps. In each heatmap, the columns represented the samples, and the rows corresponded to different chromosomes. The SNP-Freq_c_ on a logarithmic scale (e-based) was encoded by a colored bar. The largest SNP-Freq_c_ values were displayed in red, and the smallest values were displayed in blue. Chromosomes that fell into one cluster (horizontal axis) had a similar SNP-Freq_c_ between the samples. The samples were also clustered (vertical axis) based on the SNP-Freq_c_ value of each sample.

First, we generated heat maps to compare the SNP frequencies on chromosomes (SNP-Freq_c_, please refer to Formula (1) in the Materials and Methods) based on the results for the 83 single cells (Fig. 1A). We observed that the SNPs were widely distributed across 24 chromosomes and showed an enrichment on chr11 and chr17 (t-test, *p* < 2.2e-16) in both the GATK and GeMS results, while the chrY harbored much less SNPs. Similar results were observed in bulk samples, even if they still had some differences (Fig. 1B). The accumulation of SNPs on these two chromosomes, accompanied by some mutated colon cancer-related genes (as we selected for our later analysis), which potentially correlated with microsatellite instability (MSI), chromosomal instability (CIN) and chromosome translocations, may be involved in the occurrence and metastasis of colon cancer^2^,^4^,^20^. Among the genes locating on these two SNP-enriched chromosomes, some like *SOX9* and *AXIN2* were detected to harbor SNPs to different degrees. These genes might be related with colon cancer in our subsequent analyses. Second, cellular heterogeneity was found among samples for either technical or biological reasons. Although we had deleted 13 (96-83) low quality samples and trimmed the rest data with strict criteria, technical bias might still exist. Notably, single-cell samples such as SRR1003782 and SRR1003856, which had a similar amount of sequencing reads, were quite different in SNP-Freq_c_. Although the cells used were cultured cells, we speculated that cellular heterogeneity might account for these differences^14^.

**Figure 2:**
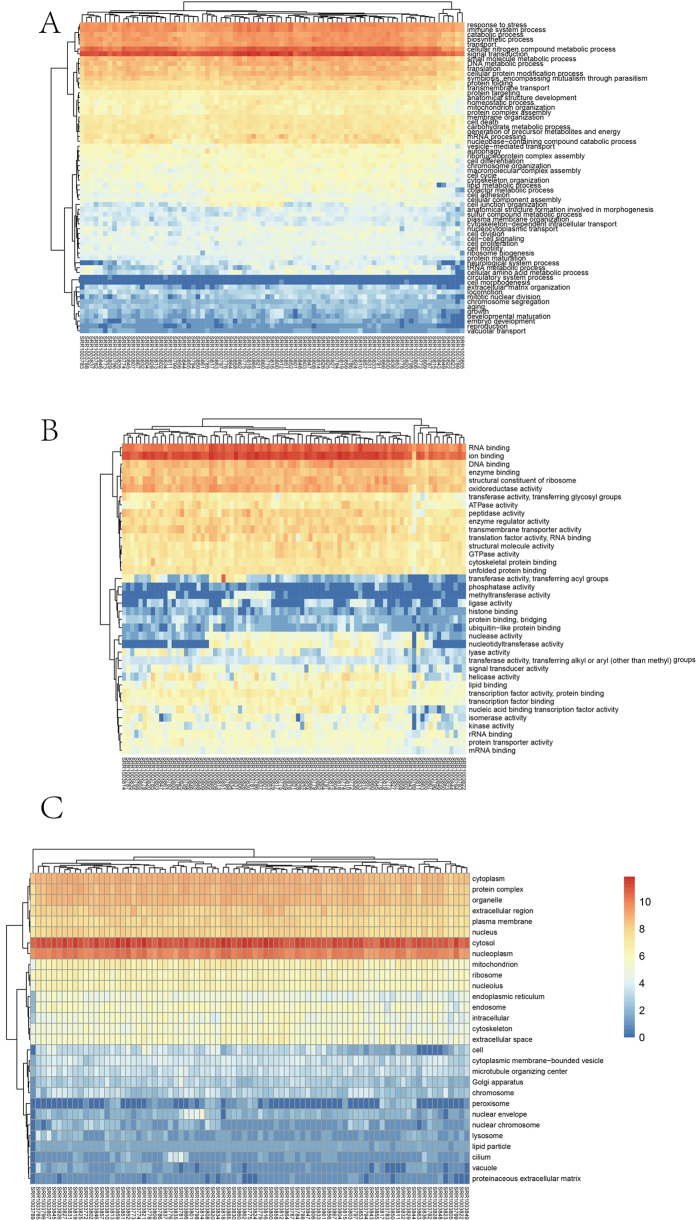
Heatmap of GO Slims for the single-cell samples based on SNP calling results by the GATK. (**A**) SNP counts for the GO Slims in the Biological Process category. (**B**) SNP counts for the GO Slims in the Molecular Function category. (**C**) SNP counts for the GO Slims in the Cellular Component category. The SNP-associated GO Slims for the three categories (Biological Process, Cellular Components, Molecular Function) among the 83 single-cells were displayed in the heatmaps. The columns represented the single-cell samples, and the rows represented the different GO Slims. Each colored cell in the heatmap represented a standardized amount of SNPs for the GO Slim on a logarithmic scale (2-based). The cells with high counts were red filled, and those with low counts were blue filled.

To identify the functions of the genes harboring SNPs, we performed GO analysis^21^ based on the GATK SNP calling results (please refer to the Materials and Methods for details). We classified these GO Slims according to three categories: biological processes (BP), cellular component (CC) and molecular function (MF). In the BP category, all GO Slims were clustered into two groups, and the GO Slims in the smaller group (Fig. 2A and Table 1) such as signal.transduction were significantly enriched (t-test *p* < 2.2e-16). In the MF category, the GO Slims were classified into three groups (Fig. 2B). The first group contained only two enriched GO Slims (iron.binding, RNA.binding) (Student’s t-test *p* < 2.2e-16), and the second group contained 4 enriched GO Slims (Student’s t-test max (*p*) < 2.2e-16) (Table 1). In the CC category, we shown that all GO Slims were clustered into two groups (Fig. 2C). The first group contained 2 enriched GO Slims and the second contained 5 enriched GO Slims. GO analysis results based on the GeMS SNP sets offered similar GO Slim list (Supplementary Table 1).

We manually selected a list of 175 potential colon cancer-related genes that had already been reported as oncogenes or tumor suppressor genes^4^,^21^,^22^,^23^,^24^,^25^,^26^,^27^,^28^ (Supplementary Table 2). We found that according to the GATK SNP sets, 12 genes harbored SNPs, some of which even shown a high “sample ratio” (defined by Formula (2) in the Materials and Methods) (Supplementary Table 3). From Supplementary Table 3, we could observe that some genes, including *PSME2, PSMD14, GNB4, PSMC1,* and *H2AFZ*, had fairly high or at least moderate sample ratio of 98.80%, 87.80%, 67.47%, 30.12% and 96.39%, respectively. When we linked these genes to their GO Slims, they were largely linked to enriched GO Slims, including nucleolus and organelle in CC, signal.transduction in BP and DNA.binding in MF. This consistency between the GO Slims and the mutated cancer-related genes might indicate the possible dysregulation of these functions in colon cancer. In the SNP results based on GeMS SNP sets, similar results were detected, reinforcing our analysis based on the GATK. Thus, we speculated that the dysregulation of gene transcription, which directly or indirectly increased the risk of colon cancer^21^, might result from variations in these cancer-related genes and damage in their associated GO Slim functions. To further investigate the importance of these cancer-related mutations, we employed pathway enrichment analysis of the SNPs by applying Wilcoxon Rank-Sum test^29^ on two groups: the cancer-related gene group and the other gene group. The results have shown that the SNP-Freq_g_s of the cancer-related gene group were remarkably higher, with a *p* < 2.2e-16, comparing with the other gene group.

We also found lists of mutated genes in different pathways. We selected colon cancer-related pathways, including the WNT, TGF-β, PI3k-AKT, p53, mismatch repair, apoptosis and MAPK signaling pathways, for our analysis. We selected all genes in each of these pathways from the Kyoto Encyclopedia of Genes and Genomes (KEGG), calculated their sample ratios and SNP-Freq_g_s, and checked the SNP enrichment for each of these pathways based on two indicators using Fisher’s test and Wilcoxon test. Then we re-ranked these pathway related genes based on sample ratios and SNP-Freq_g_s^30^ (for details please refer to the Materials and Methods). After this two-step ranking, we observed a list of highly or moderately mutated genes in each of the pathways. This list included genes such as *BMP7, MAPK1, GNB4, HSP90AB1, HSP90B1, PERP, RPA1* and *HSPA8*, which had a comparatively high SNP-Freq_g_; and genes such as *RAC2, YWHAH, JAK3, CDK4, CCNB1, ELK4,* and *DFFA*, which were moderately mutated (Table 2). Moreover, SNPs had also been detected in most of these genes according to the GeMS results, which validated our analyses based on the GATK results.

### Mutations activating tumor progression in the TGF-β signaling pathway and its crosswalks

**Figure 3:**
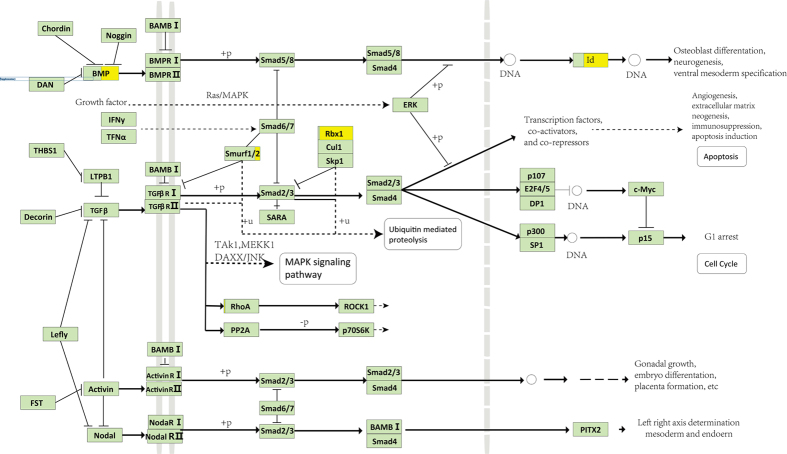
TGF-β signaling pathway and its mutated genes. The TGF-β signaling pathway and its gene interactions and mutations were displayed. The light green boxes represented SNP-free genes, and the yellow boxes represented genes harboring SNPs. For genes shown in two colors, the ratio of the yellow area indicated the sample ratio of mutations for this gene.

All of the results of the cancer-related gene and pathway enrichment analyses were based on the GATK analyses. In general, we had observed that some pathways may be dramatically altered due to gene mutations, particularly mutations in pivotal genes, which were accompanied by abnormal expression of the associated upstream or downstream genes in the pathways. One such major alteration occurred in the TGF-β signaling pathway (Fig. 3). The TGF-β signaling pathway regulates tumor progression and has either a tumor-suppressing or a tumor-promoting function, depending on the cellular context^31^. In this risky pathway, we found that 5 out of 80 genes mutated, and many of them were linked to the enriched GO Slims such as DNA.binding and nucleolus.

*RBX1*, a RING component of SCF (skp-1, cullins, F-box proteins) E3 ubiquitin ligases, mutated in 80 cells with comparatively higher expression (t-test *p* = 2.2e-16). *RBX1*, also called *ROC1*, regulates diverse cellular progresses by targeting a variety of substrates for degradation^32^. In many human cancers, *RBX1* and *RBX2* are overexpressed in multiple human tissues and are required for the growth and survival of cancer cells^33^. We suggested that there might be abnormal expression of *RBX1* since it harhored SNPs in many cells. In the same sub-pathway of TGF-β, *SMURF2* mutated in 13 cells. As it has a negative effect on the receptor of TGF-β, its mutations might affect the normal function and promote the TGF-β signaling.

Meanwhile, in 35 single cells, the tumor suppressor gene *BMP7* mutated with a high SNP-Freq_g_ value, which were about half of the SNP-Freq_g_ value for *BMP7* in bulk cancer samples. And in most of these 35 single cells, the expression of *BMP7* were not detected based on RNA-Seq data. *BMP7* is a key cytokine that exerts two divergent effects in the colon mucosa: one is to reverse the TGF-β-induced epithelial-to-mesenchymal transition (EMT) required for tumor metastasis; the other, however, is likely to associate with pejorative functions during chronic ulcerative diseases and neoplastic progression^34^,^35^. In the downstream of *BMP7, ID2* mutated in 80 cells. Aberrant *ID2* activity was reported as a direct initiation and progression of embryonal cancer^36^.

### DNA damage checkpoint mutations and the anti-apoptotic process induce possible tumor activation through the p53 signaling pathway

**Figure 4:**
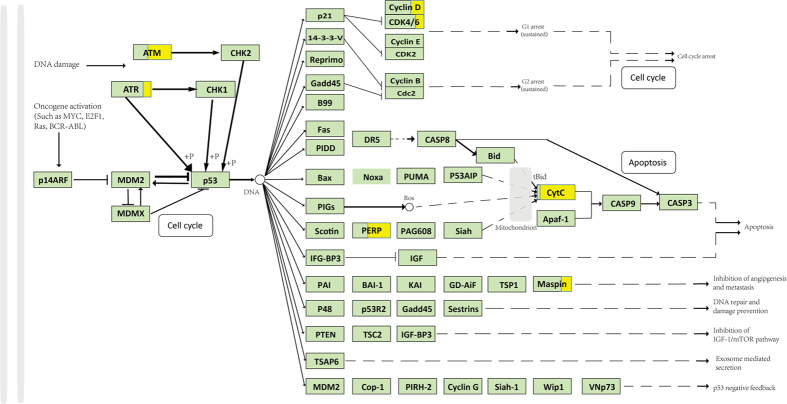
p53 signaling pathway and its mutated genes. The p53 signaling pathway and its gene interactions and mutations, along with the distribution of the expression of some important genes in the mutated and non-mutated samples were shown. Genes in light green did not contain SNPs. Genes partially in yellow contained mutations. The ratio of the yellow area of a gene indicated the sample ratio of this gene.

Potential functional variations on DNA damage checkpoint genes and some p53 downstream genes may work together to alter the p53 signaling pathway (Fig. 4). We found 6 mutated genes, and among them, *ATM, ATR, PERP, CYCS* and *CCNB1* had relative moderate sample ratios.

Two DNA damage checkpoint genes, *ATM* and *ATR*, mutated in 8 and 13 cells, respectively, with high SNP-Freq_g_ values. We observed higher *ATR* expression in cells with mutated *ATR* (t-test, *p* = 0.02335), while no clear association was shown between *ATM* expression and its mutation (Fig. 4). *CHEK1* and *CHEK2* are target genes of *ATR* and *ATM*, though no significant difference was found in the expression of these two genes between *ATM* (or *ATR*) mutated samples and *ATM* (or *ATR*) non-mutated samples, dysregulation might also occurred. These variations in checkpoint genes might have potential effects on the pivotal gene *TP53*, which is essential for apoptosis and genomic stability (Fig. 4). Upon testing the association between *TP53* expression and checkpoint gene mutations among the cells, we found that the expression of *TP53* decreased (t-test *p* = 0.001033) in cells with *ATM* mutations. We also checked the expression of *TP53*-associated genes such as *MDM2* and *MDMX* and found no significant variance among the cells^37^. In summary, these mutated checkpoint genes and their possible downstream feedbacks on *TP53* might damage the function of *TP53* in tumor suppression.

In another part of the p53 pathway, *PERP* and *CYCS* (the gene encoding *CytC*) (Fig. 4), which are all involved in apoptosis, mutated in large fraction of cell sets. *PERP*, an apoptosis gene that responds to DNA-damage, presented an 60.64% sample ratio^38^. Since *PERP* is a direct target of *TP53*, we inferred that its role in apoptosis might possibly be impaired in consequence. Moreover, mitochondria-associated genes were also mutated in a manner that would alter the apoptotic process. *CYCS*, which is an important factor in mitochondrial apoptosis, mutated in 97.59% of the cells. The defects in or even loss of the apoptosome (composed of CytC, Apaf-1 and Pro-caspase9) might be caused by *CYCS* mutations. And its role in cleaving death substrates would be further impaired^39^.

### Possible cancer-related mutations in other cancer-related pathways

Genetic alterations were also detected in some other cancer-related pathways. In the PI3K signaling and apoptotic pathways, *HSP90B1, HSP90AB1, 14-3-3* genes and *GNB4* were detected with high sample ratios and SNP-Freq_g_ values. We detected 14-3-3 protein-encoding gene mutations in *YWHAQ, YWHAE* and *YWHAB* and *YWHAZ,* with a sample ratios ranging from 84.44% to 98.80%. 14-3-3 proteins not only play essential roles in the pro-proliferative and anti-apoptotic pathways, but also participate in tumor suppression, particularly after DNA damage^40^. Although these 14-3-3 genes shown moderate expression compared with the other genes in the pathway, mutations in two pro-apoptotic downstream genes, *BAD* and *FOXO3*, as well as mutations in the pivotal upstream gene *AKT*, which seldom expressed in non-cancerous cells, suggesting potential damage in the tumor suppression activity of the 14-3-3 genes.

In the MSI pathway, *MSH3, RPA1* and *RPA3* mutated in 4.81%, 60.24%, 4.82% of samples, respectively. As confirmed by our results, these mutated genes, which could transform normal cells into mismatch repair-deficient ones, tend to accumulate functional alterations^41^. In the WNT signaling pathway, *CSNK2B, RHOA, RBX1* and some downstream cell cycle genes mutated in samples. Notably, the proteasome, which degrades β-catenin through the destruction complex (composed of *AXIN, GSK3Beta* and *APC*), contained several mutations in its constructional or functional genes, which probably led to a higher degree of free β-catenin in cells. β-catenin translocates to the nucleus and promotes the DNA replication and cell growth, thereby promoting tumor progression^42^.

### Comparison of the SNP profiles from the 96 single-cell samples with those from the bulk cancer and normal samples

We used both bulk cancer and bulk normal samples for SNP analyses in the same procedure for single-cell analysis and shown that although the SNP structure was globally consistent in the GO results and cancer-related SNP detection, the single-cell RNA-Seq data had its own advantages in delineating cell heterogeneity, which was always averaged in bulk samples.

First, the bulk cancer samples displayed chromosomal enrichment on chr17, chr19 and chr22 (two group t-test, *p* = 0.003808), overlapping part of the single-cell results (Fig. 1B). Second, the bulk cancer GO analysis results were similar to the single-cell functional analysis results, and both results also exhibited unique GO Slims (Tables 1 and 3). Compared with GO analysis results based on bulk samples, the single-cell samples presented two specific enriched groups in the MF category, including RNA.binding and DNA.binding, and the enriched GO Slims in the other two categories almost matched the single-cell list. These uniquely enriched GO Slims echoed the results of the cancer-related gene analysis, in which we found that a list of cancer-related genes were associated with these enriched GO Slims.

Comparing with the pathway analysis of the results for the bulk samples, the single-cell RNA-Seq displayed more interesting information at a higher resolution, which was unfortunately averaged in the bulk samples. Although fewer mutated genes were identified in each individual single-cell samples, similar results were obtained from the single-cell and bulk cancer samples in all pathways analyzed above. Whereas, there was also a list of genes mutated in single-cell samples but lacked mutations in bulk ones, like *ID2, RPA3, CYCS, CCNB2, CDK4* and *RAF1*. Additionally, genes such as *ID2, HSP90AB1, CYCS, CCNB1* and *RPA3* have shown a much higher SR (sample ratio) in single-cell samples. Instead, the less frequently mutated genes including *EP300, LRP6, PRKX, BMP8A, ID3, PPM1D, ATR, GNG12*, and *ZAK* were found to be more frequently mutated in the bulk cancer samples. Furthermore, some mutations were detectable even in the bulk normal sample, although they were located in the cancer-related genes or in the pathways discussed above. This might be in accordance with a similar finding that some cancer-related genes might still acquire mutations to different degrees, even in normal samples, because of factors such as individual genetic backgrounds and environmental stress^43^.

### Fusion transcript detection in the single-cell samples

**Figure 5:**
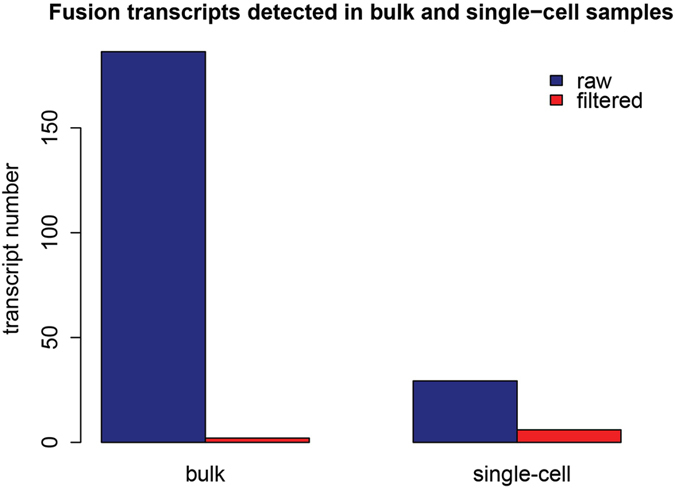
Fusion transcripts detected in bulk and single-cell samples. The number of fusion transcripts before and after filtering both the single-cell and bulk cancer samples were shown in the figure. The blue boxes represented the transcript quantity before filtering, while the red boxes referred to the transcript quantity after filtering.

Fusion transcripts have often been thought to stimulate tumorigenesis^44^. Genomic rearrangements in colorectal carcinoma, which lead to the fusion of essential genes as well as to other oncogenic events, have attracted much attention in cancer research. Here, we had identified a list of fusion genes, including some biomarkers of colon cancer. *EML4-ALK*, which is closely connected to cell growth, was found in 11 single-cell samples^45^. We also detected fusions such as *VTI1A-TCF7L2* and *PTPRK-RSPO3,* in 33 and 5 cells, respectively^20^,^46^. Additionally, a novel fusion transcript, *UBB*-*UBC*, was detected in 5.21% of the single-cell samples based on the single-cell RNA-Seq data (for details, please refer to the Materials and Methods). Given the observed elevations in UB levels in many tumor types (also observed in this study)^47^, as well as the versatility of the UB system in various cellular systems, we speculated that this *UBB*-*UBC* fusion transcript might activate or hyperactivate related functions or ubiquitination-mediated signaling pathways^48^. Cell-level heterogeneity of fusion transcripts was also apparent, possibly owing to stochastic processes or reactions to cellular stresses^49^. Based on the finding of bulk colon cancer sample, though we detected more than 6 times the number of raw fusion transcripts (186.33 fusion transcripts on average) than for single-cell samples (29.31 on average), fewer fusion transcripts were identified after filtering (2 on average, for details, please refer to the Materials and Methods) (Fig. 5), indicating that we might be able to detect more fusion transcripts with fidelity at the single-cell level.

## Conclusion

Single-cell sequencing is not only a powerful technique that can almost replicate the results of bulk sequencing, but also a tool that can reveal individual cell properties and heterogeneities that have been hidden in bulk-sample SNP analyses. This inspiring technology will help us to uncover a biological “personality” by resolving intratumor heterogeneity, tracing cell lineages and allow us to understand the mechanisms of tumorigenesis^5^,^14^. In our single-cell RNA-Seq analyses of colon cancer cells, the single-cell SNP calling results had performed well for detecting the bulk averaged genetic clues. In summary, we analyzed the SNP structure on chromosomes, employed GO analysis (which suggested potential SNP-caused malfunctions), performed cancer-related gene, pathway enrichment and fusion gene analyses. All of these experiments helped us to identify important mutated genes, fusion transcripts and disorders in pathways that are essential for our understanding of colon cancer at the single-cell level. We also implemented the same protocol for single-cell analyses on bulk samples and found that the results for the single cells were mostly consistent with the results for the bulk cancer samples. Furthermore, analyses at the single-cell level detected more subtle and important genetic clues, which might shed light on cancer, particularly for early diagnosis. Although this method still faces the dilemmas of low genome coverage, high amplification bias and a lack of suitable algorithms, some patterns such as single-cell SNP profiles and heterogeneity patterns at the single-cell level were already evident. Future integration of these analytical results with rational experiments for assessing key gene mutations at the bulk level could push forward the functional validation of key mutations that were discovered only at the single-cell level. Accordingly, single-cell sequencing is on its way to becoming a powerful method for scientists to explore genetic diversity in cell populations.

## Materials and Methods

### RNA-Seq data for the single-cell and bulk samples

HCT116 is a human colon cancer cell line (of male origin) and can be obtained from the American Type Culture Collection (ATCC; CCL-247). RNA-Seq data for 96 single HCT116 cells were downloaded from National Center for Biotechnology Information (NCBI) for colon cancer SNP analysis^14^ (BioProject ID: 222225). These HCT116 cells were maintained in RPMI-1640 medium and were then sorted into and frozen in standard 96-well plates. These single-cell samples were all biological replicates obtained from a microfluidic platform. cDNA synthesis was then performed in an MJ Thermocycler according to the supplier’s protocols before the Illumina libraries were constructed. Sorted single-cell libraries and bulk libraries were sequenced 1 × 50 bases on Illumina HiSeq 2000 with replicate libraries of each type in the same lane^14^.

For the bulk colon cancer samples, we downloaded RNA-Seq data for 4 bulk cancer samples from the HCT116 cell line from NCBI (BioProject ID: 222225) (Supplementary Fig. 9). Total RNA was prepared from ~1 million HCT116 cells using a dynal mRNA Direct kit. Replicates of the bulk prep were made into cDNA libraries using two different methods, with two replicates for each method. cDNA was then synthesized, and the sorted bulk cancer libraries were also sequenced using an Illumina Hiseq 2000 sequencer. For the bulk normal samples, the RNA-Seq data (GSM1010974) were downloaded from the Reference Epigenome Mapping Center^21^.

### Single-cell RNA-Seq alignment and SNP calling

The single-cell RNA-Seq raw data were processed through stringent quality control using Parallel-QC^50^, a parallel quality control computational engine for NGS data. In this quality process, we only considered uniquely mappable reads with a trim quality value of no less than 20 and a threshold ratio (the minimum percentage of bases that must meet the trim quality value) of 0.8 as our candidates. We also set the GC content to range from 0.4 to 0.6 and used tag sequence file to assist in the cleaning process. For alignment, we mapped the RNA-Seq data to the refMrna (hg19 Gene Reference, downloaded from http://hgdownload.cse.ucsc.edu/downloads.html#human) using Spliced Transcripts Alignment to a Reference (STAR)^51^. We selected STAR because it is faster than other methods and it could improve mapping by running a second mapping pass (for which we have applied on single-cell datasets). To maintain compatibility with the following three SNP callers, we made the pre-filtered BAM using Picard (a set of tools in Java for working with NGS data in the BAM format) and the GATK for filtering. In SNP calling step, we called SNPs using three SNP callers, the GATK, SAMTools and GeMS for the sake of higher accuracy and sensitivity since no specific SNP calling algorithm has been customized for single-cell RNA-Seq data.

**Figure 6:**
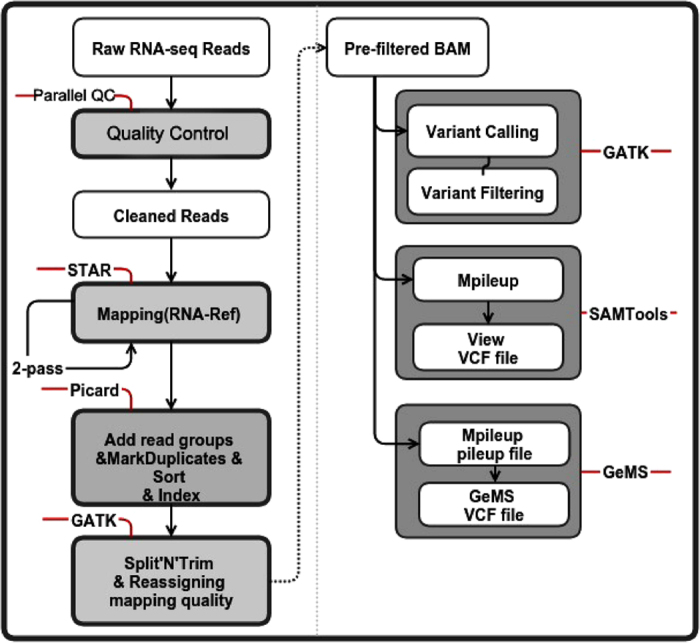
SNP calling pipeline based on the RNA-Seq data. The different types of SNP calling data were shown in the white boxes (without grey boxes outside). The steps using different tools and their detailed manipulations were shown in the gray boxes wrapped with font lines on the left of the figure. On the right half of the figure, the steps that belong to certain SNP callers were framed in the gray box. The words tagged on the red lines were the tools that we used in this SNP calling step.

1. In the GATK, we used HaplotypeCaller, which performs better than UnifiedGenotyper for variant calling (options: -dontUseSoftClippedBases -recoverDanglingHeads -stand_call_conf 20.0 -stand_emit_conf 20.0; we set -stand_emit_conf 20 specially for RNA-Seq data), and VariantFiltration for the variant filtering (we set -window 35 -cluster 3 specific for RNA-seq data; other options: -filterName FS -filter “FS > 30.0” -filterName QD -filter “QD < 2.0”).
2. In SAMTools, we used mpileup and view to call SNPs (mpileup options: -DSugf; view options: -Ncvg).
3. In GeMS, we first employed mpileup to transform BAM into the pileup format (options: -q 20 -Q 17 -Bsf) using SAMTools and then ran GeMS (because GeMS is based on Genotype Model Selection, we set –n to 0.7 to ensure confident identification; there was an additional step added in to filter the *p* of the Dixon test to remove SNPs with *p* values below 0.05) (Fig. 6).

### SNP Filteration

Owning to the limitation of single-cell RNA-Seq, we performed further filtrations to obtain suitable SNP sets.

1. We first deleted samples with low sequencing depth and coverage.
2. We then reserved a SNP if it was nonsynonymous, otherwise we filtered it out.
3. For each SNP, we further trimmed by only retaining ones which was covered by least 5 reads in a sample.
4. Since the examination of heterogeneities among single-cell samples was one of the focuses, we ensure that SNP coverage (indicating how many samples covered a SNP position) was more than 95% of samples. It is worth noticing that ensuring SNP coverage in 100% of samples was technically possible, but that might result in high false negative results, so here 95% has been used as the threshold.

### Evolutionary stress analysis

To explore the potential evolutionary stress on the orthologs, we calculated their Ka/Ks (omega) values and associated them with the GO Slim terms.

1. We performed an all-versus-all BLAST based on all human genes (obtained from refMrna) to filter the paralogs (selection option: identity >70%, e-value > 1e-10).
2. We employed Inparanoid^52^ to obtain the GO terms of each orthologous gene set and then employed in-house scripts to retrieve the GO Slim terms for each GO term (For details about this part, please refers to subsection SNP GO analysis below).
3. We filtered the GO Slim terms with less than 10 orthologs as well as the orthologs themselves. Orthologs with an expression rate of less than 95% (indicating the expression of this ortholog was detected in 95% of samples) were also eliminated, as were the ones with mutations in less than 5% of the single-cell samples.
4. We employed MUSCLE^53^ alignment of each ortholog, and the results were generated in phylip format. We ran Phyml^54^ to construct the evolutionary tree of each ortholog (We set –f m to calculate base frequencies using a maximum likelihood ratio. We also set –o tlr for better optimization, as well as –b 100, followed by PAML analysis^55^ (codeml was used, with the following options: seqtype = 1, model = 0, Nssites = 0) to calculate the Ka/Ks value for each orthologous gene.

### SNP enrichment on chromosomes

To find an overview of the SNP distribution and the possible enrichments of SNPs on chromosomes, we normalized the SNP frequency on each chromosome.

The SNP frequency on each chromosomes was calculated using the following formula:


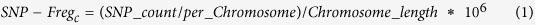


in which SNP_count/per_Chromosome is the amount of the SNPs detected on one chromosome, and Chromosome_length is the length of this chromosome. The factor 10^6^ is applied to the denominator to leverage the SNP-Freq_c_ values for a fair and easy comparison.

### SNP GO analysis

Each gene was associated with at least one GO term, which reveals the function of the gene. Thus, if a gene harbors SNPs, its functions may be altered; accordingly, the more SNPs found in a gene, the higher the probability that this gene might be altered. Here, we regarded the SNP counts on a gene as the occurrence of this gene in one sample, as well as the counts of GO terms that were associated with the gene. Thus, we could examine which function was significantly overlapped with the SNPs, indicating there were possible alternations in the function. The following detailed steps were used:

1. Converted all RefSeq IDs to gene symbols in all SNP result files (obtained using the GATK and GeMS).
2. Employed in-house scripts to match genes to their GO terms based on the human gene association file (http://geneontology.org/page/download-annotations).
3. Using a generic GO Slim term file, performed a back trace on GO terms to obtain the GO Slim of each SNP (GO Slims have provided a broad overview of the ontology content without the detail of specific fine grained terms (http://geneontology.org/page/download-ontology); thus, it is suitable for summarizing the possible functions that the SNPs might alter).
4. Classified all GO Slims associated with SNPs into three categories (Biological Process; Cellular Component; Molecular Function) using in-house scripts for the following analysis.
5. Produced a heatmap of the 96 single-cell sample GO Slims for each category and utilized the K-means clustering methods^56^ to classify the GO Slims into several groups and then analyzed the GO Slim enrichment for the respective groups by Student’s t-test.

### Analysis of the overlapping SNPs on cancer-related genes

We detected SNPs on cancer-related genes to examine whether some genes harbored SNPs or even exhibited SNP enrichment, which indicates their possible correlations with colon cancer progression.

1. We obtained a list of colon cancer-related genes that had been reported in many studies (Supplementary Table 2) and examined the overlapping SNPs in each sample. The GO Slims of the mutated genes were also added in Supplementary Table 2.
2. We calculated the sample ratio of each cancer-related gene that harbored SNPs as follows:
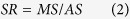
in which SR is the sample ratio, MS represents the number of mutated samples with at least one SNP detected, and AS represents the number of all samples being considered.
3. To further address the importance of the cancer-related gene groups, we classified all genes into two groups, the cancer-related gene group and the other gene group. We manually deleted some outliers and normalized the data to meet the requirement of homogeneity of variance across cell samples. We performed the Wilcoxon Rank-Sum test^29^ to analyze the SNP enrichment (*p* threshold set to be 0.05) of the cancer-related gene group compared to other groups.
4. To identify which genes contributed to the significance of the cancer-related gene group, we first simulated the density distribution of SNP-Freq_g_ values of the other gene group for each gene in a cell using the bootstrapping sampling method. Then, we tested whether the SNP-Freq_g_ of this gene fell in a unilateral 95% confidence interval. If the SNP-Freq_g_ was larger than the value of this 95% interval, we determined that this SNP-containing gene was enriched in this sample compared with the other genes. Otherwise, we did not treat the gene as enriched in SNP. After testing the enrichment of all 175 genes in each sample, we obtained a 96*175 matrix, in which there were only two values in each column, 1 and 0. A value of 1 represents enriched, and 0 represents a gene that was not enriched for that cell.
5. Based on the matrix mentioned in (4), we have selected enriched genes from all 175 cancer-related genes, so that each of the enriched genes has its status as “enriched in SNP” in more than 50% of all 96 single-cells.

### Pathway Enrichment Analysis

We selected a few important colon cancer-related pathways and analyzed their SNP distributions to comprehend the effects of the SNPs on the destruction or enhancement of these pathways.

1. We first determined the gene list in each pathway and calculated the SNP frequency (SNP-Freq_g_) of each gene using in-house scripts. The normalized SNP-Freq_g_ was calculated by the following formula:
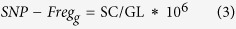
in which SNP-Freq_g_ represents the SNP Frequency in a gene, SC is the SNP count for this gene in a sample, and GL represents the length of this gene. The factor 10^6^ is applied to the denominator to leverage the SNP-Freq_g_ values for a fair and easy comparison.
2. We then validated if pathways we have chosen were enriched in SNPs, by comparing SNP Frequencies for pathway-related gene set against non-pathway-related gene set using Fisher’s test.
3. Furthermore, we assessed differences in sample ratios between the pathway-related gene set against non-pathway-related gene set using Wilcoxon test.
4. To account for expression dependence, we applied bootstrapping to randomly sample a subset of non-pathway-related genes (same amount as pathway-related genes) and tested if the results of SNP-Freq**g** for this subset is significantly enriched compared to the expressions of subset genes for pathway-related genes.
5. We have ranked the genes in each pathway based on the values of SNP-Freq_g_ and then the sample ratio. Because we observed that the genes with high sample ratios were mainly the genes with a high SNP-Freq_g_, we selected the genes using a 50% sample ratio threshold. Additionally, we manually selected the genes with a high SNP-Freq_g_ but a comparatively lower sample ratio and the genes which are documented to be possible cancer-related genes based on the KEGG pathway. All of the selected genes were tested to identify SNP enrichment compared with the other genes in the pathway using Student’s t-test.

### Fusion gene analysis

We employed deFuse^57^ with strict criteria (details as follows) to filter the fusion transcripts from the single-cell RNA-Seq data.

1. We first ran deFuse to obtain the preliminary filtered fusion transcripts from each cell. Here, we obtained 29.31 transcripts for single-cell samples on average, and 186.33 for bulk samples on average.
2. We then deleted the transcripts with one of the partner annotated with a ribosomal gene.
3. We then discarded the transcripts with at least one of the partners located in an intron or intraexonic sequence.
4. We also deleted transcripts if one of the genes had multiple partners in this cell.
5. Finally, we filtered out transcripts with partners located less than 100 kbp apart, which might denote for intrachromosomal fusion genes.
6. After these steps of filteration, single-cell samples obtained 5.96 transcripts on average, while bulk samples only retained 2.

## Additional Information

**How to cite this article**: Chen, J. *et al*. Single-cell SNP analyses and interpretations based on RNA-Seq data for colon cancer research. *Sci. Rep.* **6**, 34420; doi: 10.1038/srep34420 (2016).

## Supplementary Material

Supplementary Tables

Supplementary Figures

## Figures and Tables

**Table 1:** Enriched GO Slims among the single cells based on the SNP calling results using the GATK.

**Biological Process**	small.molecule.metabolic.process** *P* < 2.2e-16
	cellular.nitrogen.compound.metabolic.process**
	signal.transduction**
	transport**
	biosynthetic.process**
	response.to.stress**
	immune.system.process**
**Cellular Component**	Cytosol** *P* < 2.2e-16
	Nucleoplasm**
**Molecular Function**	RNA.binding** *P* < 2.2e-16
	ion.binding**
	DNA.binding**
	enzyme.binding**
	oxidoreductase.activity**
	structural.constituent.of.ribosome**

The enriched GO Slims were those with significantly higher SNP counts (for genes in these GO terms) among all GO Slims. Note: GO Slims that do not have a listed *p* value shown have the same *p* value as the terms listed above.

**Table 2:** Important mutated genes in the pathway analysis.

Pathway	Highly mutated genes and genes with high sample ratios
WNT signaling pathway	*CSNK2B* (81); *RHOA* (1); *RBX1* (80)
TGF-β signaling pathway	*BMP7* (35); *SMURF2* (13); *ID2* (80); *RBX1* (80); *RHOA* (1)
PI3K-AKT signaling pathway	*HSP90B1* (2); *HSP90AB1* (79); *GNB4* (27); *YWHAQ* (70); *EIF4B* (76); *HSP90AA1* (80); *YWHAE* (82); *YWHAZ*(80); *RPS6* (77); *YWHAB* (82)
p53 pathway	*PERP* (57); *CYCS* (81); *SERPINB5* (21); *ATR* (13); *CCNB1* (21); *CDK4* (13);
MAPK signaling pathway	*HSPA8* (1); *CRKL* (36); *ELK4* (29); *RPS6KA3* (33); *RAC2* (35); *STMN1* (81); *FOS* (80)
Apoptosis signaling pathway	*DFFA* (35); *ATM* (8); *PRKX* (13); *CYCS* (81); *NFKBIA* (20);
Mismatch repair pathway	*RPA1* (50); *MSH3* (4); *RPA3* (80); *SSBP1* (60)

Genes with high sample ratios or with high SNP-Freq_g_ values were biologically important. In each bracket, the number referred to the sample ratio of the gene calculated based on the GATK results.

**Table 3:** Enriched GO Slims among the bulk cancer cells.

**Biological Process**	biosynthetic.process** *p* = 5.646e-05
	cellular.nitrogen.compound.metabolic.process** *p* = 8.73e-05
	cellular.protein.modification.process** *p* = 0.002881
	immune.system.process** *p* = 0.0006364
	response.to.stress** *p* = 3.487e-05
	signal.transduction** *p* = 0.001852
	small.molecule.metabolic.process** *p* = 4.26e-05
	transport** *p* = 0.001235
**Cellular Component**	cytoplasm** *p* = 9.458e-07
	cytosol** *p* = 1.221e-12
	nucleoplasm** *p* = 3.493e-08
	nucleus** *p* = 3.252e-06
	organelle** *p* = 1.887e-06
	plasma membrane** *p* = 0.0004033
	protein complex** *p* = 3.295e-07
**Molecular Function**	ion.binding** *p* = 0.001278

GO Slims were classified into three categories: Biological Processes, Cellular Components, and Molecular Function. The enriched GO Slims and their *p* values (Student’s t-test) of enrichment compared with the other GO Slim groups were shown. Note: GO Slims without *p* values shown had the same *p* value as the GO Slims listed immediately above.
