# Supplementary material for: Single-cell SNP analyses and interpretations based on RNA-Seq data for colon cancer research
Source: Sci Rep. 2016 Sep 28;6:34420. doi: 10.1038/srep34420 (PMC5039670; doi:10.1038/srep34420)
Supplement: Supplementary Figures [file srep34420-s2.doc]

**Supplementary information for “Single-cell SNP analyses and interpretations based on RNA-Seq data for colon cancer Research”**

Jiahuan Chen^1,2,3^, Qian Zhou^2^, Yangfan Wang^3^, Kang Ning^1,2^,*

^1^ Key Laboratory of Molecular Biophysics of the Ministry of Education, College of Life Science and Technology, Huazhong University of Science and Technology, Wuhan, Hubei, China

^2^ Bioinformatics Group of Single Cell Center, Shandong Key Laboratory of Energy Genetics and CAS Key Laboratory of Biofuels, Qingdao Institute of Bioenergy and Bioprocess Technology, Chinese Academy of Sciences, Qingdao, Shandong, China 266101

^3^ College of Marine Life, Ocean University of China, Qingdao, Shandong, China 266000

*Corresponding author

E-mail: ningkang@hust.edu.cn

**Supplementary Figures**


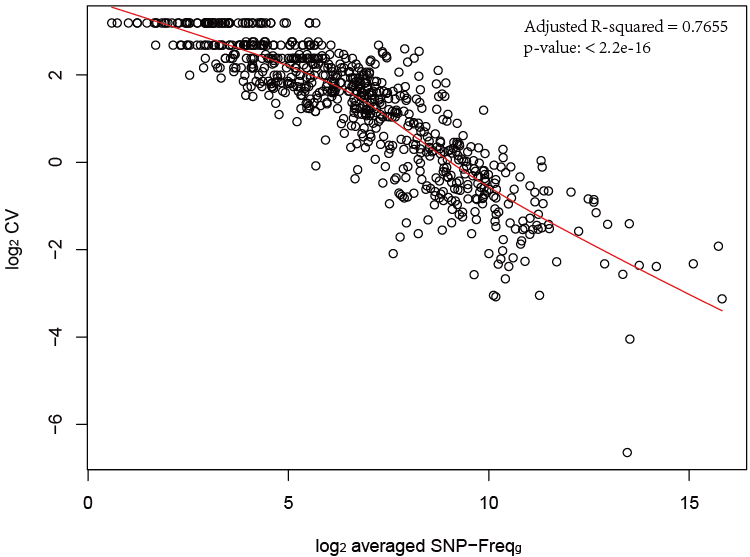


**Supplementary Figure 1. correlation analysis for averaged SNP-Freqg and CV of SNP-Freqg.**

"Averaged" means that the values were averaged for all single-cells used.


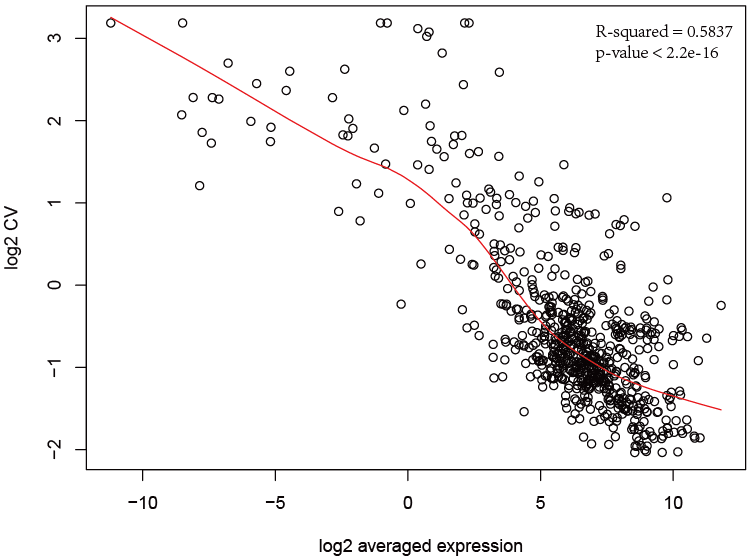


**Supplementary Figure 2. correlation analysis for averaged expression and coefficient of variation (CV) of expression.**


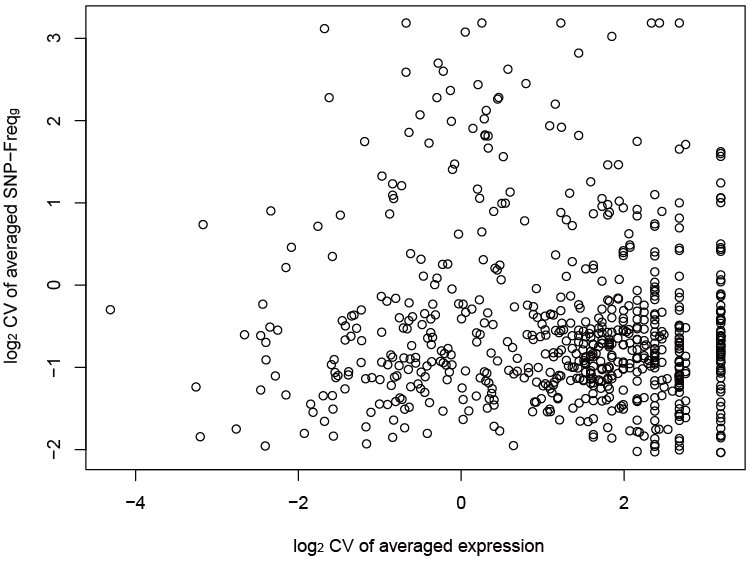


**Supplementary Figure 3. correlation analysis for CV of averaged expression and averaged SNP-Freqg.**


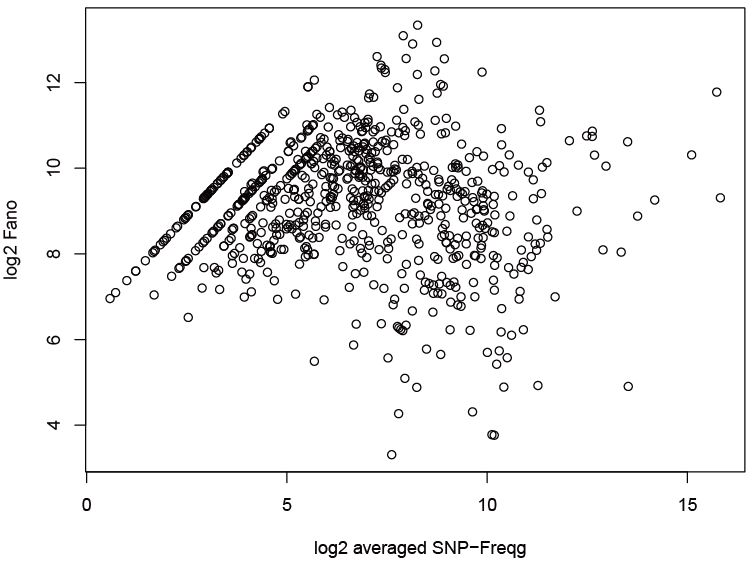


**Supplementary Figure 4. correlation analysis for averaged SNP-Freqg and Fano factor.**


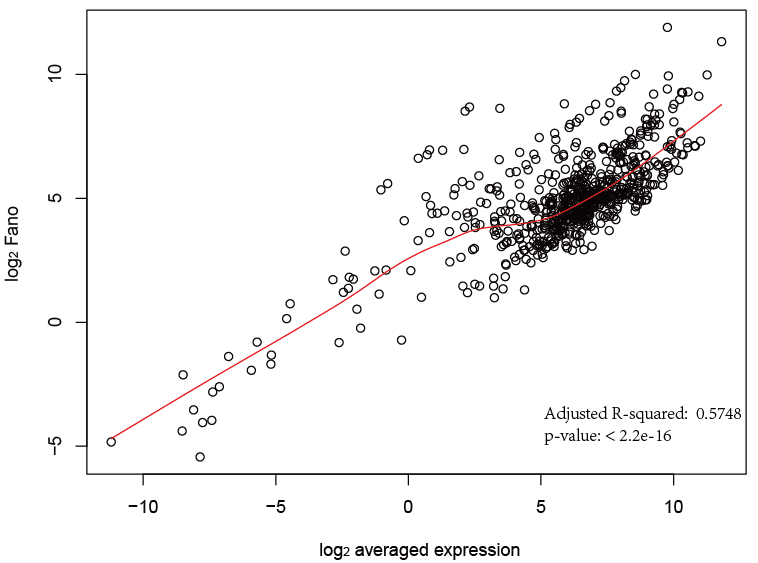


**Supplementary Figure 5. correlation analysis for averaged expression and Fano factor.**


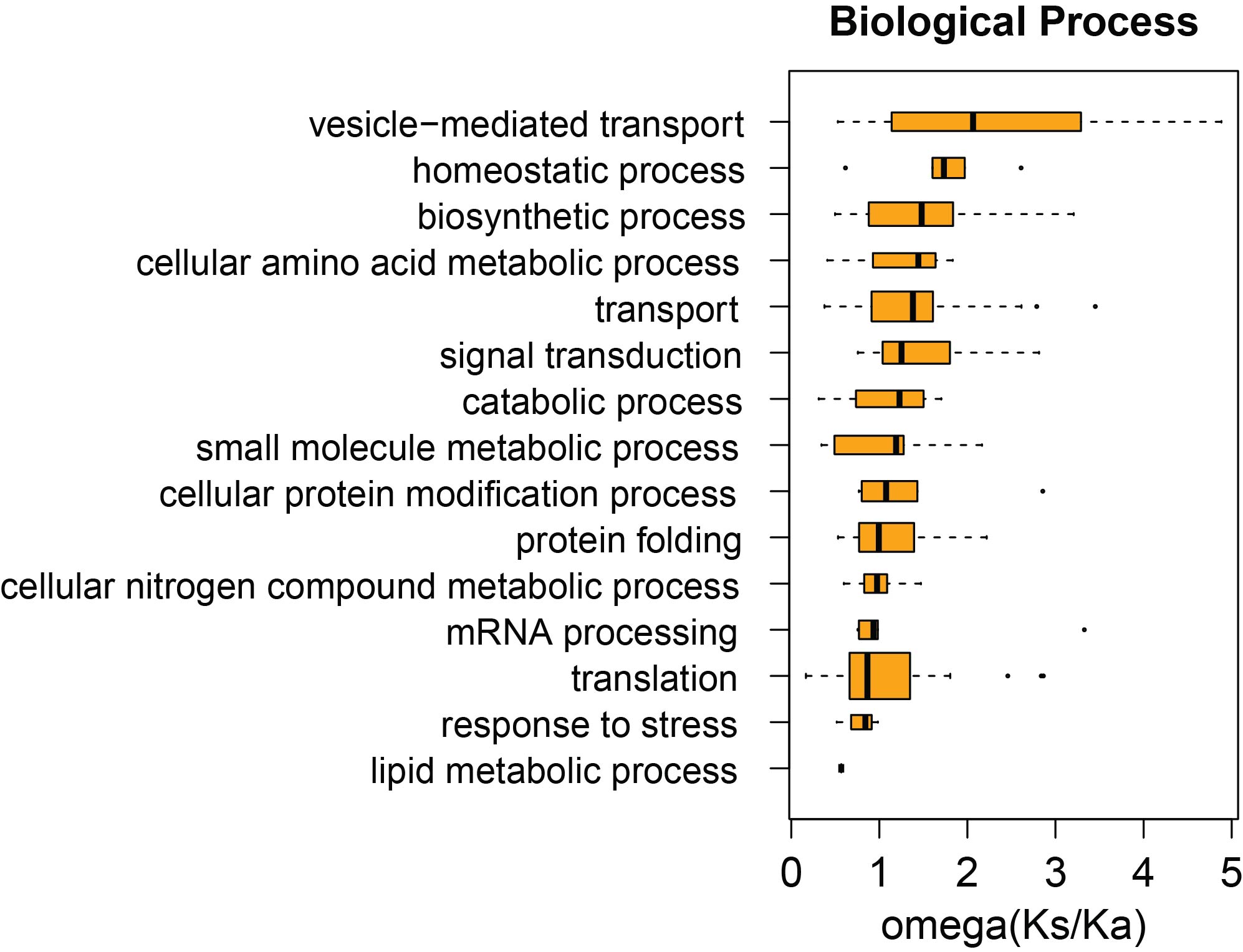


**A**


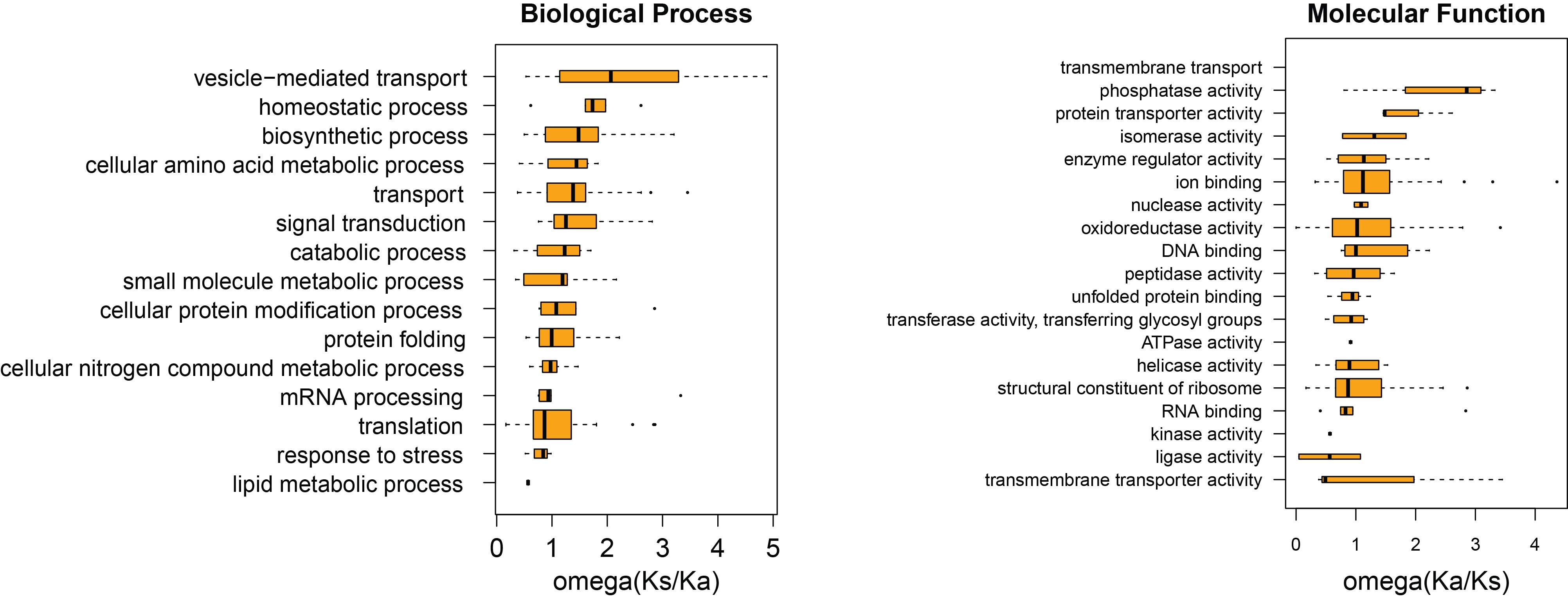


**B**


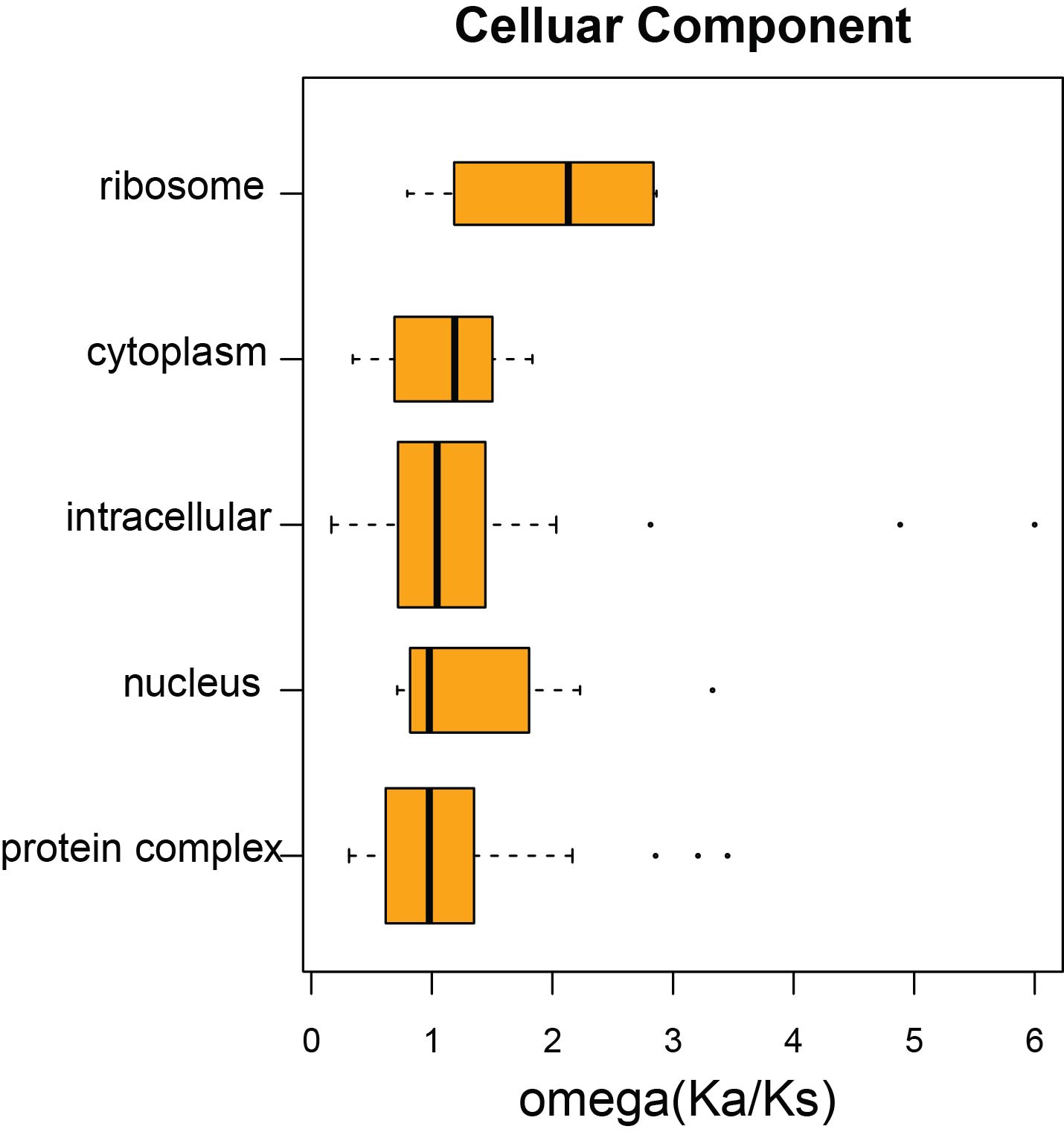


**C**

**Supplementary Figure 6 | Evolutional stress on GO Slims** | The evolutionary stress on certain GO Slim terms based on omegas (Ka/Ks) were shown. Omega (or Ka/Ks) value was the ratio of the number of nonsynonymous substitutions per non-synonymous site (Ka) to the number of synonymous substitutions per synonymous site (Ks), which could be used as an indicator of selective pressure acting on protein-coding gene. Comparison of orthologous genes with high (>1) Ka/Ks values that were under positive selection, while Ka/Ks value less than 1 was considered as purify selection. **(A)** represented results for GO Slims in the BP category. **(B)** represented results for GO Slims in the MF category. **(C)** referred to the results for GO Slims in the CC category. Each GO Slim contained a list of orthologous genes with their computed Ka/Ks values. All GO Slims in each sub-figure were ordered based on the median of each box.


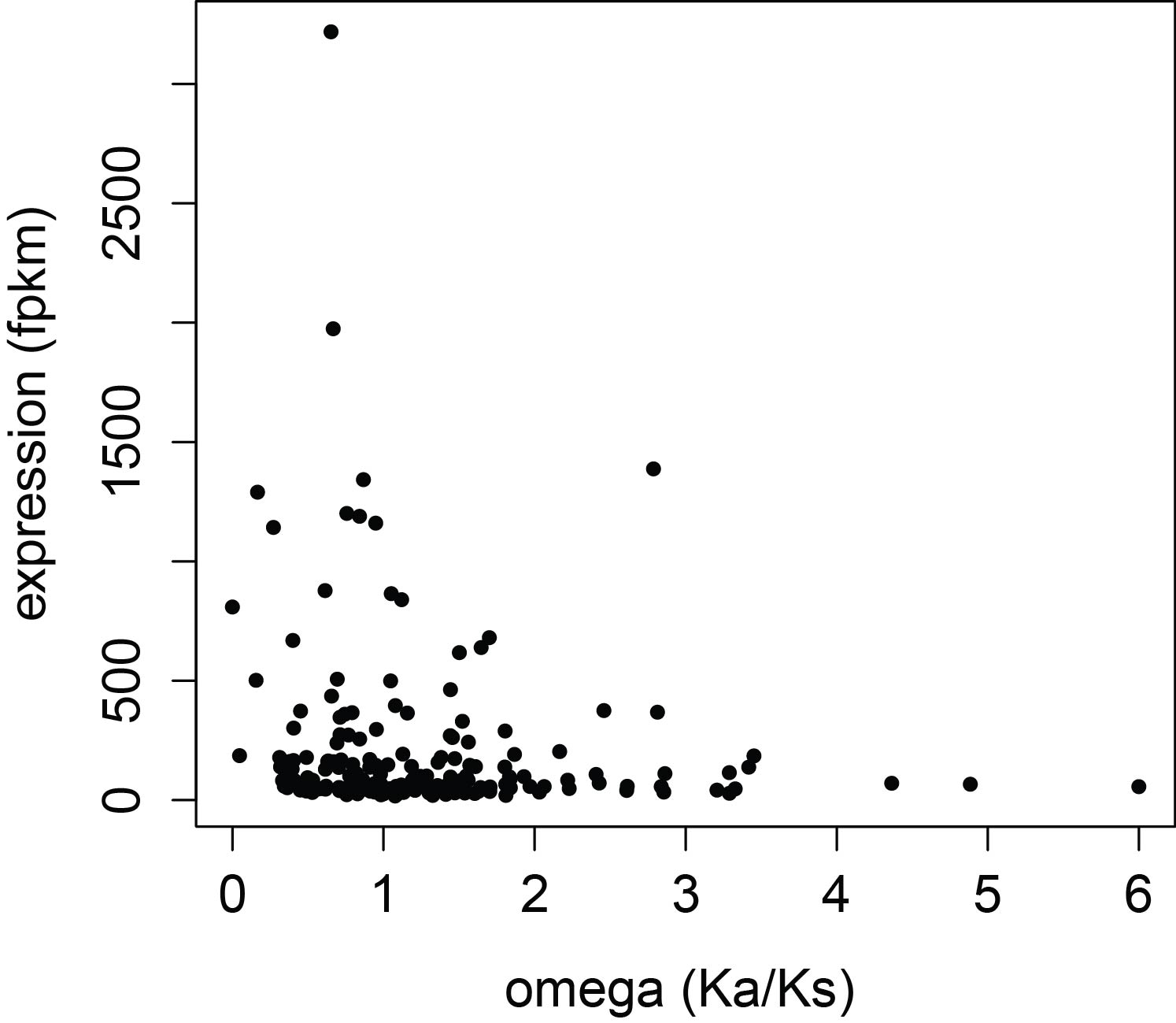


**Supplementary Figure 7 | Correlations between omega and related gene expression |** The relationships between gene expressions and gene Ka/Ks values were displayed, in which each dot represented an orthologous gene.

A


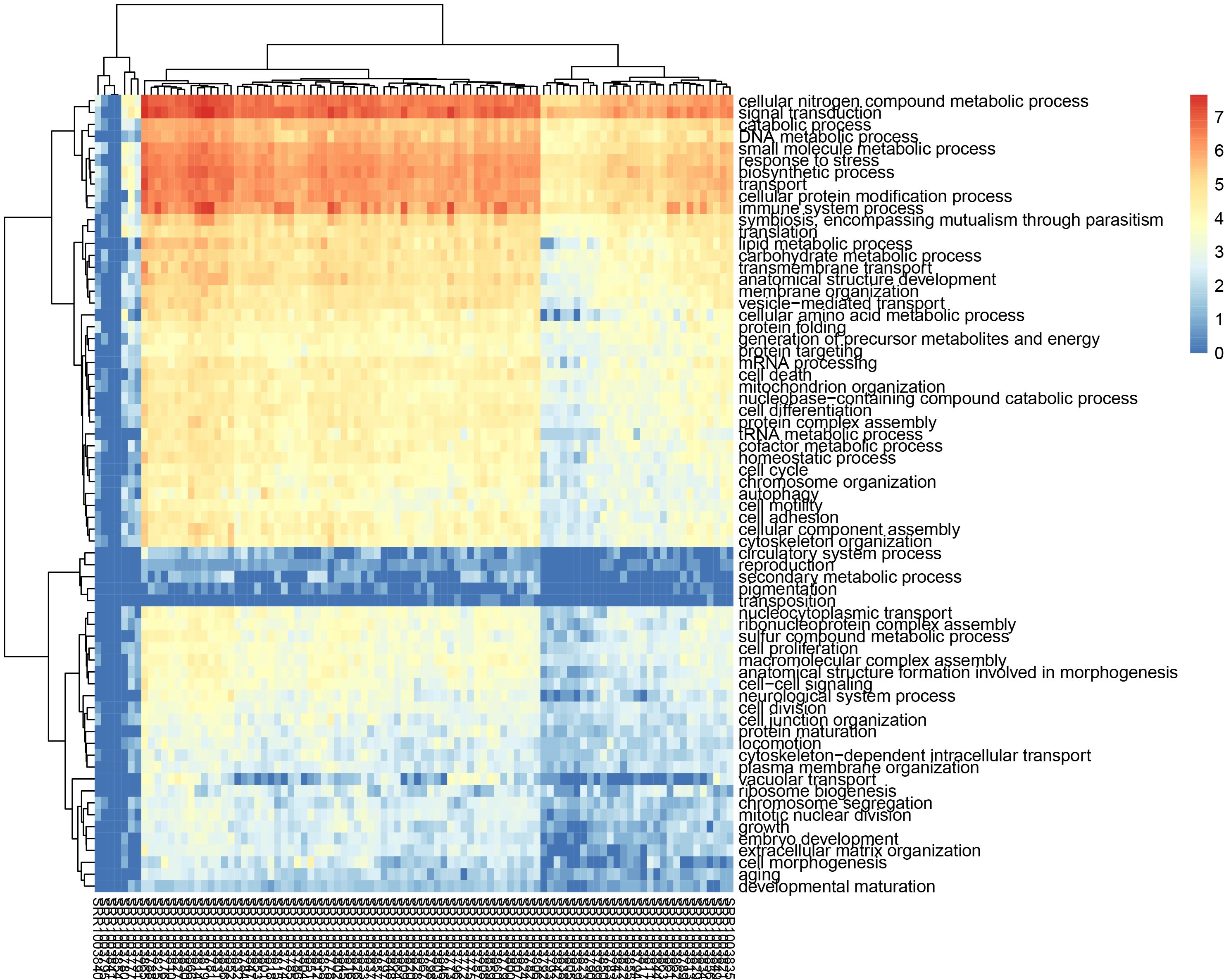


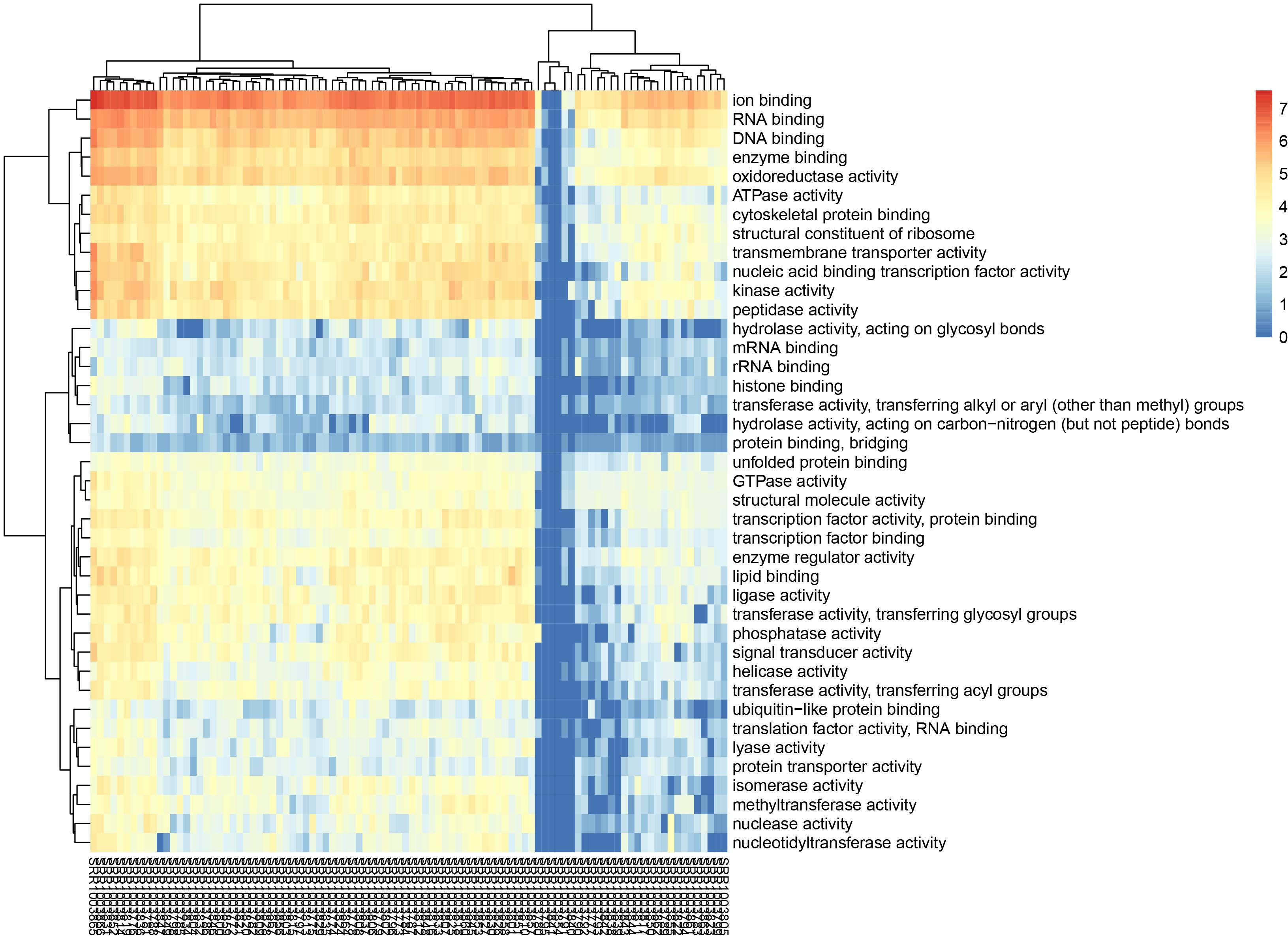


B

**
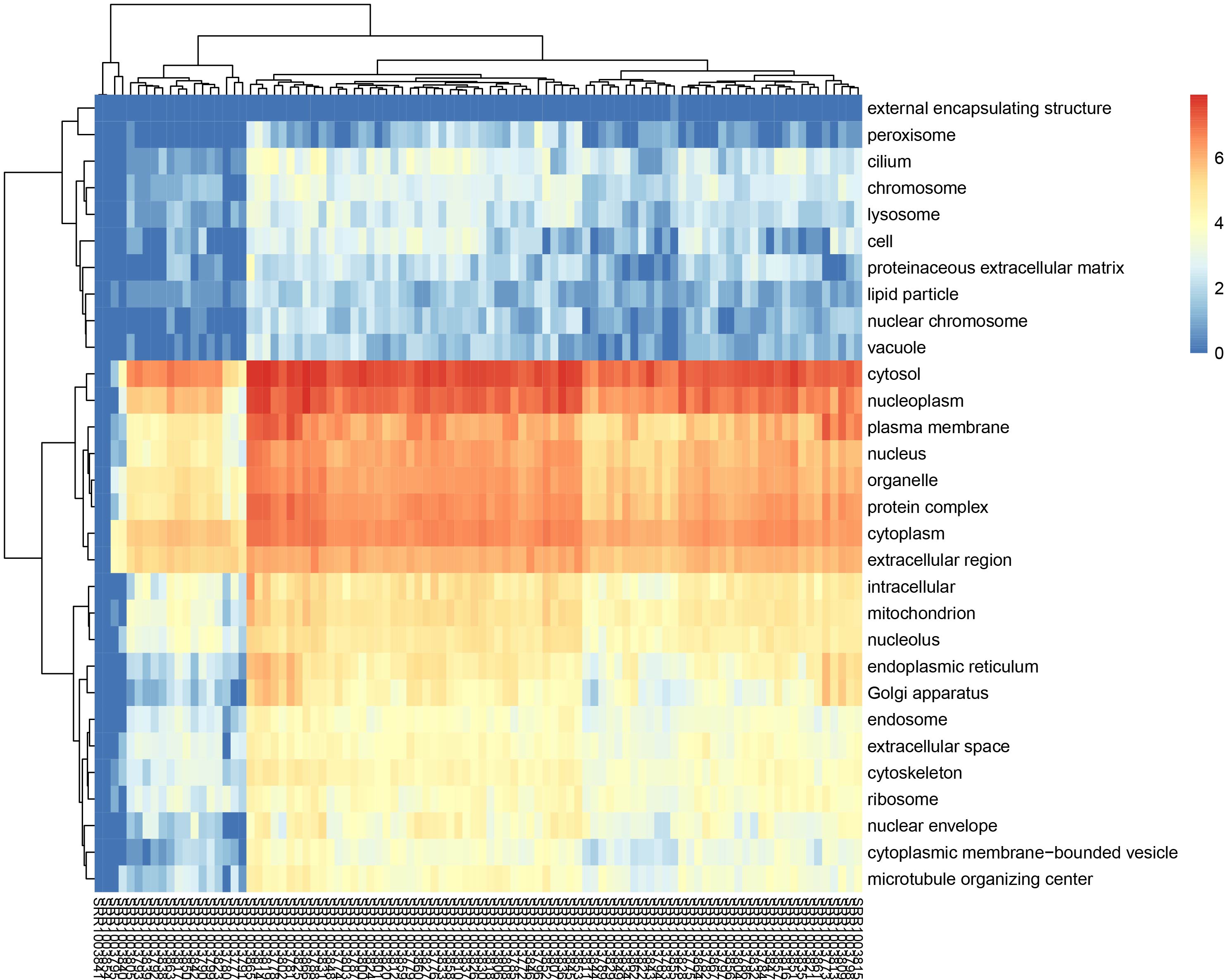
**

C

**Supplementary Figure 8 | Heatmap of GO Slims of single-cells based on SNP calling result by GeMS |** (**A**) SNP counts for GO Slims in the category of Biological Process. (**B**) SNP counts for GO Slims in the category of Molecular Function. (**C**) SNP counts for GO Slims in the category of Cellular Component. Columns represented single-cell samples, and rows represented GO Slims which contained genes with identified SNPs. In each heatmap, each colored cell represented standardized count of the GO Slim on a logarithmic value (2-based). Both GO Slims and samples were clustered on x-axis and y-axis, respectively.


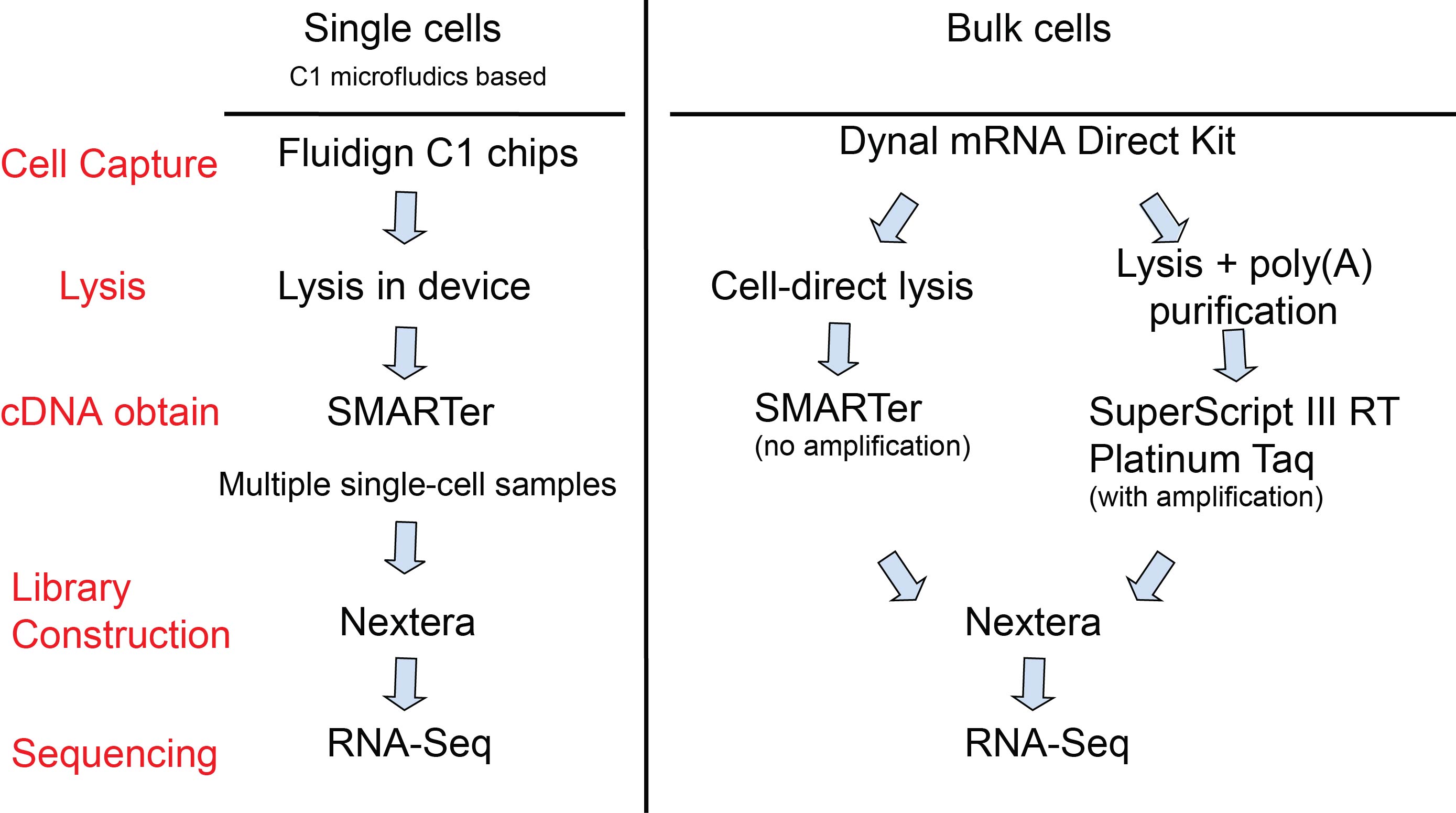


**Supplementary Figure 9 | Schematic of RNA-Seq samples preparation |** This was a concise procedure of RNA-seq sample preparation for both bulk cancer and single ones. The red words at left were the recapitulation of each step. For detailed methods, please referred to[^1^](#_ENREF_1).

**References**

1 Wu, A. *R. et a*l. Quantitative assessment of single-cell RNA-sequencing methods*. Nature metho*d**s** 11, 41-46 (2014).
